# Adherence to the 24-hour movement behavior guidelines and depression risk among older adults from the United States

**DOI:** 10.1186/s44167-024-00071-7

**Published:** 2025-01-09

**Authors:** Astrid N. Zamora, Arjan S. Walia, Abby C. King

**Affiliations:** 1 Department of Epidemiology and Population Health, Stanford University School of Medicine, 1701 Page Mill Rd, Palo Alto, CA 94304 USA; 2 Stanford Prevention Research Center, Department of Medicine, Stanford University School of Medicine, Palo Alto, CA 94304 USA

**Keywords:** Aging, MVPA, Older adults, Sedentary behavior, Sex differences, Sleep duration, 24-hour movement behavior

## Abstract

**Background:**

While recent studies, primarily among Asian cohorts, have linked adherence to 24-hour movement behavior (24-HMB) guidelines with improved mental health—some of which show sex differences—few studies have explored these relationships among older adults from the United States.

**Methods:**

National Health and Nutrition Examination Survey data from 2011-2018 were examined in 2,812 older adults (≥ 65years). Those considered adherent to 24-HMB guidelines had a sleep duration of 7–8 h./night, moderate-vigorous physical activity (MVPA) ≥ 150 min/wk., and sedentary behavior (SB) < 8 h./day. Sleep duration, SB, and MVPA were self-reported, with SB and MVPA obtained from the validated Global Physical Activity Questionnaire. Depression was measured using the Patient Health Questionnaire (PHQ-9), with a score of ≥ 10 indicating depression. Logistic regression was used to evaluate overall and sex-stratified associations between non-adherence to all three behaviors, combinations of two behaviors, or individual behavior guidelines, with odds of depression, adjusted for putative confounders.

**Results:**

Among the full sample, non-adherence to all three 24-HMB guidelines was associated with 1.7 [95% confidence interval (CI):1.1, 3.1; *p* = 0.02] higher odds of depression versus those that adhered to all three behaviors. After sex stratification, the association only persisted among males [OR = 2.5 (95% CI:1.1, 5.4); *p* = 0.02]. Within the overall sample, higher odds of depression were observed for those who did not adhere to the SB + sleep duration guidelines and the sleep duration + MVPA guidelines. Sex-stratified findings revealed that associations only remained significant in males. While in the overall sample of older adults, non-adherence to the sleep duration guideline was associated with 2.1 (95% CI:1.4, 3.3; *p* = 0.001) higher odds of depression compared to those that adhered to the guideline.

**Conclusions:**

Results provide evidence of associations between non-adherence to 24-HMB and higher odds of depression, specifically in older males, suggesting a potential sex-specific effect that warrants further investigation. Future studies using longitudinal designs are needed to confirm these findings and explore the mechanisms underlying these associations.

## Background

Presently, nearly 62 million older adults (defined here as adults ≥ 65 years) reside in the United States (US) and comprise 20% of the total population [1]. Projections indicate that by 2054, the older adult demographic will increase to 84 million, representing nearly one-fourth of the total population in the US [1]. Depression is the most common psychological problem among the aging population [2]. A recent report from the Centers for Disease Control (CDC) indicated that 14.2% of older adults had been diagnosed with depression [3]. However, known barriers to reporting depression symptoms, such as depression-related beliefs, symptom severity, absence of family history, fear of being prescribed medication, and concerns about medical record confidentiality [4], could result in an older adult not being properly diagnosed. Thus, the reported prevalence of depression among this demographic may be an underestimate, and the actual prevalence may be much higher, particularly among older adults susceptible to loneliness and other cognitive changes associated with depression [5]. Furthermore, experiencing depression in late age not only diminishes the quality of life in the older population but also correlates with deteriorations in physical health and cognitive and social functioning, along with posing other health risks that may increase the risk of mortality [6]. Thus, it is crucial to identify risk factors for depression among older adults.

Numerous studies have supported the independent association of depression and depressive symptoms with decreased physical activity [7, 8], sedentary lifestyles [9–11], and poor sleep patterns [12, 13]. However, sedentary behavior, physical activity, and sleep health behaviors intersect [14], and taking a siloed, single-behavior approach neglects the fact that movement behaviors are co-dependent. Moreover, due to their collective influence on each other and potential cumulative impact on depression, it is imperative to examine the interplay between these movement behaviors rather than just focusing on one behavior alone [15]. The Canadian Society for Exercise Physiology developed the 24-hour movement guidelines (24-HMG) for children and youth [16] and more recently adapted guidelines for older adults aged 65 years or older, irrespective of gender, cultural background, or socioeconomic status [17].

While numerous studies have examined associations between adherence to the 24-HMB and mental health status of adolescents [18, 19], and young adult populations [20], primarily within the global context of Asia, few studies have examined these associations in older adult populations. A recent study revealed that adhering to the 24-HMG was closely related to the mental health of older Chinese adults [21], while another study published in 2024 found that those who more closely adhered to these guidelines had lower odds of depression [22]. While the aforementioned studies have reported that associations between physical activity, sedentary behavior, and mental health can depend on the domain of these behaviors [21, 22], the distribution of these behavioral domains may vary significantly between Chinese and US older adults due to cultural, environmental, and lifestyle differences [23]. As a result, it is unclear whether the findings observed in Chinese older adults can be generalized to the US older adult population, highlighting the need to examine these relationships within the unique context of US older adults. Additionally, a previously described cross-sectional study of a Chinese sample reported sex differences in the relationship between combinations of meeting 24-HMB recommendations and mental health, with results being stronger in females compared to males [21]. While evidence on sex differences remains limited, we propose that similar patterns may exist among US older adults, warranting investigation.

To the authors’ best knowledge, there are no existing studies that have examined adherence to 24-HMB guidelines in relation to depression among a nationally representative sample of US older adults or explored differences in these associations according to sex. To fill this gap, this cross-sectional study used National Health and Nutrition Examination Survey (NHANES) data to explore the relationship between adherence to the 24-HMB guidelines and the odds of depression among older US adults - the fastest-growing demographic in the country.

## Methods

### Study population

**Fig. 1:**
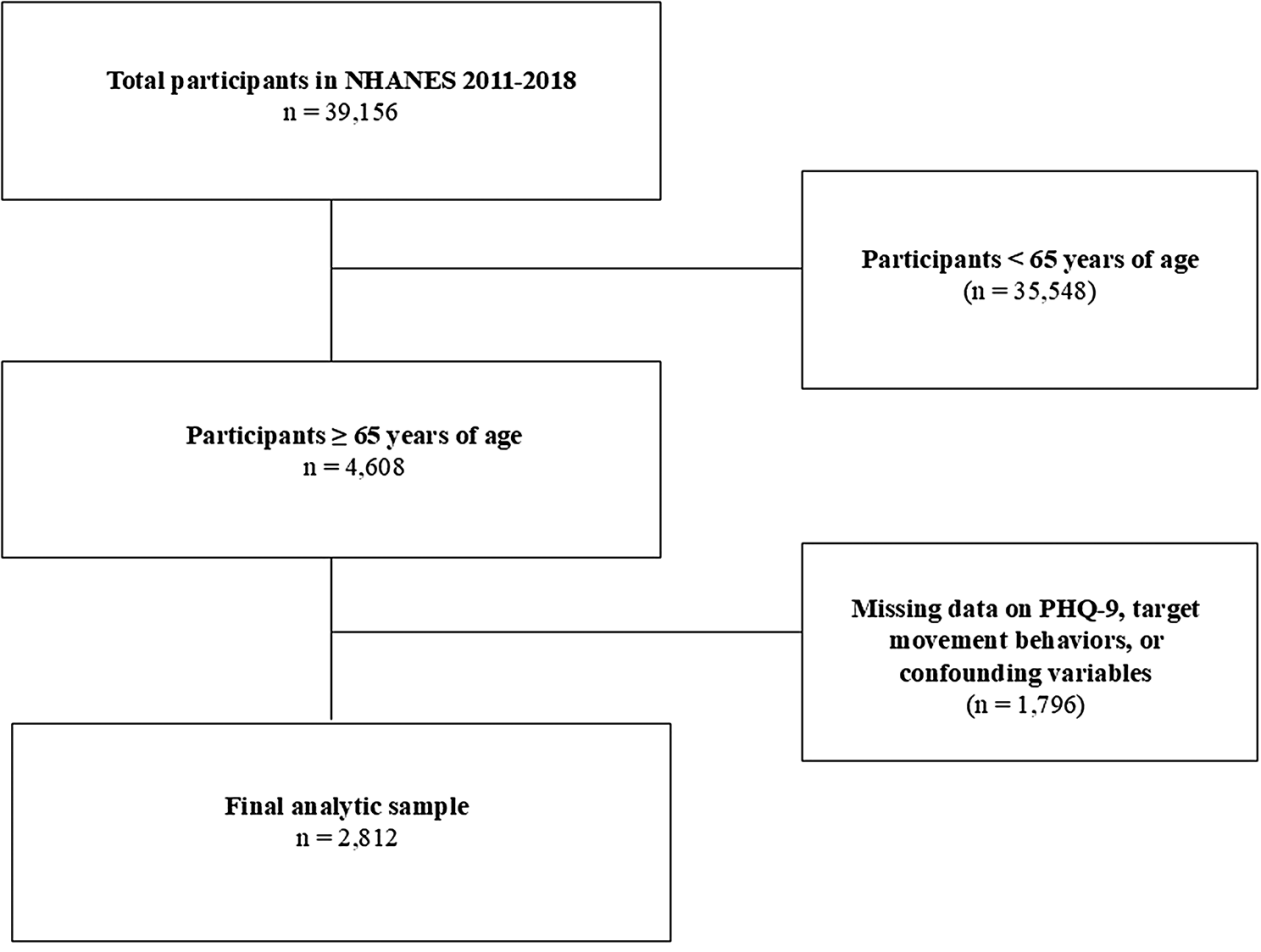
Schematic flow diagram of inclusion and exclusion criteria

Participants in the current investigation were adults ≥ 65 years of age with complete data for all variables of interest from four consecutive NHANES cycles: 2011–2012, 2013–2014, 2015–2016, and 2017–2018 [24]. We excluded any participants that were 1) < 65 years of age; 2) were missing data on the target movement behaviors, including self-reported sleep duration, sedentary behavior, and MVPA; 3) were missing data on the Patient Health Questionnaire-9 (PHQ-9); and 4) were missing data on any potential covariates that could serve as putative confounders. All other participants comprised the analytic sample, with the final eligible sample comprising 2,812 participants (See Fig. 1 for flow diagram).

NHANES has approvals from the Ethics Review Board of the CDC National Center for Health Statistics, and all participants provided written informed consent at the time of data collection. As the present investigation utilized publicly available de-identified NHANES data, it was considered exempt from human subject research procedures by the Stanford University Institutional Review Board.

### Calculation of 24-hour movement behavior guidelines

The Canadian Society for Exercise Physiology criteria for adhering to 24-HMB guidelines for older adults [17] was used, which included obtaining 7–8 h of sleep per night, ≥ 150 min per week of MVPA, and < 8 h per day of sedentary behavior. To create the binary 24-HMB variable, participants who met all three guidelines received a score of either 1 (adhering to all three) or 0 (not adhering to all three). The following variables were created to capture combinations of adhering to two guidelines: (1) sleep and sedentary behavior, (2) sleep and MVPA, and (3) sedentary behavior and MVPA. Participants were given a score of 1 if they met guidelines for combined behaviors or a score of 0 if they did not meet guidelines for both. Additionally, binary variables were generated for each individual guideline: sleep duration, MVPA, or sedentary behavior, with 0 denoting those that did not adhere to the individual guideline and 1 representing those that adhered to the guideline.

Time spent in sedentary behavior was obtained from the following question: “The following question is about sitting at work, at home, getting to and from places, or with friends, including time spent sitting at a desk, traveling in a car or bus, reading, playing cards, watching television, or using a computer. Do not include time spent sleeping. How much time do you usually spend sitting on a typical day?” Sedentary behavior was operationalized as a continuous variable reflecting minutes per day in all analyses.

Average sleep duration was abstracted from the following question from NHANES cycles 2011–2014: “How much sleep do you usually get on weekdays or workdays (number of hours)?” While for NHANES cycles 2015–2018 the question asked participants: “number of hours usually sleep on weekdays or workdays.” The two variables were merged to create one sleep duration variable.

Total minutes per week of MVPA was estimated via the Global Physical Activity Questionnaire (GPAQ), which includes questions related to daily, leisure time, and sedentary activities. The GPAQ has been validated and deemed reliable for physical activity monitoring in various studies, including NHANES [25, 26]. During the interview, participants reported the frequency and duration of their occupational, transportation, and leisure-time physical activities over a typical week. Specifically, for transportation, they indicated how many days they walked or bicycled for at least 10 min and the number of minutes spent doing so each day. Leisure-time and occupation-related questions addressed engagement in both vigorous and moderate-intensity activities and similarly had participants report by frequency and duration during a typical week. This allowed us to calculate weekly minutes of MVPA by summing the total vigorous and moderate minutes. Notably, as per the current US National Physical Activity Guidelines [27], for calculation purposes, one minute of vigorous physical activity was equated to two minutes of moderate-intensity physical activity. Overall, MVPA minutes per week were computed by summing minutes from occupational, transportation, and leisure-time activities, following established methods [28].

### Assessment of depression

Depression was assessed using the PHQ-9. The PHQ-9 is a self-report depression screening tool that is based on nine items reflective of major depressive disorder in the Diagnostic and Statistical Manual of Mental Disorders, Fourth Edition [29]. It measures depression severity and includes questions related to the frequency of depressive symptoms experienced over the past two weeks. Each question is rated on a scale from “0” (not at all) to “3” (nearly every day), with the sum of scores ranging from 0 to 27, with higher scores indicating greater severity of depression. Scores of 10 or greater were used to denote the presence of depression (yes or no) [29].

### Sociodemographic covariates

Age (years), sex (male and female), and race/ethnicity information were obtained from the NHANES demographic file. Of note, the label gender is used in the NHANES dataset; however, because the response options pertain to sex, the term sex is consistently used throughout this manuscript.

Race/ethnicity was categorically operationalized as follows: non-Hispanic Asian, non-Hispanic Black, Latinx/Hispanic (including Mexican-American), non-Hispanic White, while participants that identified as any other race/ethnicity or identified as multi-racial were collapsed into the other or multi-racial group. Assessment and classification for marital status, poverty income ratio, educational attainment, smoking status (never, current, former), and body mass index (BMI) have been previously described elsewhere [30].

### Statistical analysis

Appropriate NHANES sample survey weights were used to account for unequal probabilities of selection, oversampling, and non-response in the NHANES survey, as recommended by the National Center for Health Statistics in all analyses.

First, the authors examined differences in depression status based on participant characteristics and adherence to the 24-HMB guidelines. This analysis included comparisons between those adhering to all three guidelines, those not adhering to any of the guidelines, and those adhering to various combinations of guidelines (as previously described above). In accordance with NHANES statistical recommendations [31], means and proportions were calculated using PROC SURVEYMEANS and PROC SURVEYFREQ. The Rao-Scott Chi-Square test was employed for binary and categorical variables to assess differences, while the weighted t-test was used for continuous variables. The data are presented as means and standard errors for continuous variables and as frequencies and percentages for categorical variables, all based on sampling weights.

Associations between adherence or non-adherence to the 24-HMB guidelines and odds of depression were examined using logistic regression models. Model 1 was unadjusted and aimed to quantify the crude association between 24-HMB guidelines and odds of depression. Model 2 included covariates selected based on a *priori* knowledge of the association between target movement behaviors and depression in other populations [21, 32]. These included age, sex, poverty income ratio, race/ethnicity, marital status, education level, smoking history, and BMI [21, 22, 32–35]. In addition, given existing knowledge showing that 24-HMB influence each other, we adjusted for any missing target movement behavior that was not the main exposure of interest in each model. For example, when combined adherence to sleep duration and MVPA guidelines was the exposure of interest, we adjusted for sedentary behavior. For all regression models, the odds ratios (OR) and corresponding 95% confidence intervals (CIs) are reported.

Additionally, subgroup analyses stratified by sex were conducted to examine whether there were differences in associations between 24-HMB guidelines and odds of depression according to sex. Statistical tests for this exploratory investigation were two-sided, with statistical significance set at *P* < 0.05. All statistical analyses were conducted using SAS 9.4 (SAS Institute Inc., Cary, NC).

## Results

The analytic study sample included 2,812 participants with a mean (SE) age of 71.9 (0.17) years, with 51.4% identifying as female and 5.1% classified as being depressed. A full description of participant characteristics by depression status is shown in Table 1. Among those with depression, 72.3% identified as non-Hispanic White (vs. other racial/ethnic groups) (*P* = 0.002), and 48.7% were former smokers (*P* = 0.003). In addition, among those with depression, nearly three-quarters (74.6%) were not living in poverty (*P* = 0.004), while more than half (52.2%) were classified as being obese (defined as a BMI ≥ 30 kg/m^2^) *(P* < 0.0001). When examining differences in adhering to 24-HMB guidelines and varying combinations of the guidelines, we found that among those with depression, only 12.5% adhered to the 24-HMB guidelines compared to 20.4% of those without depression that adhered to the 24-HMB guidelines (*P* = 0.02). Finally, a greater proportion of those with depression did not adhere to any of the three guidelines compared to those without depression (13.2% vs. 4.9%; *P* = 0.004). We observed similar differences in those adhering to individual guidelines according to depression status (See Table 1).

**Table 1:** Participant characteristics according to depression status (*N* = 2,812)

		Overall sample	Not depressed (*N* = 2,644)		Depressed (*N* = 168)
Age, years, mean (SE)		71.9 (0.17)	71.9 (0.18)		71.2 (0.45)
	*P*			0.14	
Sex, N (%)					
Male		48.6	49.1		39.1
Female		51.4	50.9		60.9
	*P*			0.06	
Race/ethnicity					
Latinx/Hispanic		6.3	6.0		11.7
Non-Hispanic White		80.2	80.6		72.3
Non-Hispanic Black		7.0	6.9		9.5
Non-Hispanic Asian		3.8	3.9		1.6
Other race/ethnicity or multi-racial		2.7	2.6		4.9
	*P*			**0.002**	
Smoking history, N (%)					
Never		49.1	49.9		34.6
Current		8.0	7.6		16.7
Former		42.8	42.5		48.7
	*P*			**0.003**	
Educational attainment, years, N (%)					
< 9		4.4	4.2		8.6
9–11		7.4	7.1		13.6
12		22.4	22.4		22.5
≥ 12		65.7	66.3		55.3
	*P*			**0.01**	
Marital status, N (%)					
Never married		3.3	3.1		5.7
Married/living with a partner		65.1	65.8		51.4
Divorced/widowed/separated		31.6	31.1		43.9
	*P*			0.06	
Poverty-income ratio, N (%)					
No poverty (score ≥ 1.0)		84.7	85.3		74.6
Any poverty (score < 1.0)		15.3	14.7		25.4
	*P*			**0.004**	
Body mass index, kg/m^2^, N (%)					
Underweight (< 18.5)		1.5	1.5		1.1
Healthy weight (18.5–24.9)		25.6	25.4		30.3
Overweight (25.0–29.9)		37.3	38.5		16.4
Obese (≥ 30)		35.5	34.6		52.2
	*P*			**< 0.0001**	
Adhering to 24 h-movement behavior guidelines					
No		80.0	79.6		87.5
Yes		20.0	20.4		12.5
	*P*			**0.02**	
Adhering to at least two guidelines					
No		38.2	37.9		43.4
Yes		61.8	62.1		56.6
	*P*			0.33	
Adhering to at least one guideline					
No		5.3	4.9		13.2
Yes		94.7	95.1		86.8
	*P*			**0.004**	
Adhering to sedentary behavior and sleep duration					
No		53.9	53.1		67.1
Yes		46.1	46.9		32.9
	*P*			**0.005**	
Adhering to sedentary behavior and MVPA					
No		66.7	66.9		63.7
Yes		33.3	33.1		36.3
	*P*			0.54	
Adhering to sleep duration and MVPA					
No		77.6	77.1		87.5
Yes		22.4	22.9		12.5
	*P*			**0.005**	
Adhering to sleep duration guideline					
No		45.2	44.2		64.0
Yes		54.8	55.4		36.0
	*P*			**< 0.0001**	
Adhering to sedentary behavior guideline					
No		15.8	15.6		17.8
Yes		84.2	84.4		82.2
	*P*			0.62	
Adhering to MVPA guideline					
No		62.5	62.5		62.2
Yes		37.5	37.5		37.8
	*P*			0.95	

ABBR: hr/night: hours per night; MVPA: moderate-vigorous physical activity; min/wk.: minutes per week; N: sample size; NH: Non-Hispanic; SE: standard error; All *P* values obtained from the Rao-Scott χ^2^ test; **Note: Boldface indicates statistical significance (***p* < *0.05)*

### Associations between non-adherence to 24-HMB guidelines and odds of depression

Table 2 presents results from multivariate logistic regression analyses examining how non-adherence to the guidelines was associated with odds of depression in the entire sample. Model 1 revealed that compared to those who adhered to the 24-HMB guidelines those who did not adhere had nearly two times higher odds [OR (95% CI) = 1.8 (1.0, 3.1); *P* = 0.03] of depression. After adjustment, the odds of depression were slightly attenuated [OR (95% CI) = 1.7 (1.1, 3.1); *P* = 0.02]. Results from other models revealed significant associations between non-adherence to the combined sedentary behavior (SB) and sleep duration guidelines with odds of depression and a separate association between non-adherence to the combined sleep duration and MVPA guidelines with odds of depression. To illustrate, compared to those who adhered to the SB and sleep duration guidelines, those who did not adhere to the guidelines had 1.8-times higher odds of depression [OR (95% CI) = 1.8 (1.1, 2.8); *P* = 0.01). At the same time compared to those who adhered to the sleep duration and MVPA guidelines, those who did not adhere to the guidelines had 1.9-times higher odds [OR (95% CI) = 1.9 (1.1, 3.4); *P* = 0.02] of depression. Finally, for individual guidelines, results demonstrated that compared to those who adhered to the sleep duration guidelines, those who did not adhere to this guideline had 2.1-times higher odds of depression [OR (95% CI) = 2.1 (1.4, 3.3); *P* = 0.001). No statistically significant associations were observed between not adhering to either the SB or MVPA individual guidelines with odds of depression in the overall analytic sample.

**Table 2:** Non-adherence to the 24-hour movement behavior guidelines and odds of depression among older adults (*N* = 2,812)

	Model 1		Model 2	
	OR (95% CI)	*P*	OR (95% CI)	*P*
	Adhering to all three guidelines			
Adhering to guidelines for all three movement behaviors (REF)				
Not adhering to guidelines for all three movement behaviors	1.8 (1.0, 3.1)	**0.03**	1.7 (1.1, 3.1)	**0.02**
	Adhering to two guidelines			
Adhering to sedentary behavior and sleep duration guidelines (REF)				
Not adhering to sedentary behavior and sleep duration guidelines	1.8 (1.2, 2.8)	**0.008**	1.8 (1.1, 2.8)	**0.01**
Adhering to sedentary behavior and MVPA guidelines (REF)				
Not adhering to sedentary behavior and MVPA guidelines	0.9 (0.5, 1.4)	0.55	0.8 (0.5, 1. 3)	0.30
Adhering to sleep duration and MVPA guidelines (REF)				
Not adhering to sleep duration and MVPA guidelines	2.1 (1.2, 3.6)	**0.009**	1.9 (1.0, 3.3)	**0.03**
	Adhering to individual guidelines			
Adhering to sleep duration guideline (REF)				
Not adhering to sleep duration guideline	2.2 (1.5, 3.4)	**0.003**	2.1 (1.4, 3.3)	**0.001**
Adhering to sedentary behavior guideline (REF)				
Not adhering to sedentary behavior guideline	1.2 (0.6, 2.2)	0.62	1.3 (0.7, 2.4)	0.46
Adhering to MVPA guideline (REF)				
Not adhering to MVPA guideline	0.9 (0.5, 1.6)	0.95	0.8 (0.5, 1.4)	0.49

Model 1: unadjusted; Model 2: adjusted for age, gender, race, marital status, education level, poverty income ratio, smoking history, and BMI as well as target movement behaviors that are not the exposure of focus (i.e. for sleep duration + MVPA guidelines, we further adjusted for sedentary behavior); ABBR: MVPA: moderate-vigorous physical activity; REF: reference; **Note: Boldface indicates statistical significance (***p* < *0.05)*

### Sex stratified results of the associations between non-adherence to 24-HMB guidelines and odds of depression

Table 3 presents results from our sex-stratified analyses examining non-adherence to 24-HMB and depression risk. Results provide evidence of potential sex differences, with most associations persisting only for males. After sex stratification, the fully adjusted model revealed that compared to males who met the all three movement behavior guidelines, those who did not adhere to the 24-HMB guidelines had 2.5-times higher odds of depression [OR (95% CI) = 2.5 (1.1, 5.4); *P* = 0.02), while this association was not statistically significant for females [OR (95% CI) = 1.0 (0.4, 2.3); *P* = 0.98]. A similar trend was observed for other combinations of guidelines. For instance, not adhering to the combined SB and sleep duration guidelines was significantly associated with 2.1-times higher odds of depression in males [OR (95% CI) = 2.1 (1.1, 4.2); *P* = 0.03], while a similar pattern was observed for females [OR (95% CI) = 1.5 (0.8, 2.9); *P* = 0.17], albeit the association was not statistically significant. Similarly, not adhering to the combined sleep duration and MVPA guidelines was associated with 2.7-times higher odds of depression in males [OR (95% CI) = 2.7 (1.2, 6.3) *P* = 0.02], but not significantly associated for females [OR (95% CI) = 1.1 (0.5, 2.6); *P* = 0.73]. Finally, the association between not adhering to the individual sleep duration guideline and higher odds of depression remained statistically significant for both males [OR (95% CI) = 2.1 (1.1, 4.2) *P* = 0.03] and females [OR (95% CI) = 2.0 (1.1, 3.7); *P* = 0.03], suggesting no difference in this association according to sex. However, a marginally significant association was observed in females, where non-adherence to the combined SB and MVPA guidelines was associated with lower odds of depression [OR (95% CI) = 0.5 (0.3, 1.0); *P* = 0.05], suggesting a potential detrimental association for females that adhered to those guidelines. In contrast, no significant association was observed in males [OR (95% CI) = 1.3 (0.7, 2.7); *P* = 0.43], though the direction of the association trended toward non-adherence being associated with higher odds of depression.

**Table 3:** Adhering to the 24-hour movement behavior guidelines and odds of depression stratified by sex

	Males (*N* = 1,513)		Females (*N* = 1,299)	
	Model 1	Model 2	Model 1	Model 2
	OR (95% CI); p	OR (95% CI); p	OR (95% CI); p	OR (95% CI); p
	Adhering to all three guidelines			
Adhering to guidelines for all three movement behaviors (REF)				
Not adhering to guidelines for all three movement behaviors	2.8 (1.3, 6.0); p = **0.01**	2.5 (1.1, 5.4); p = **0.02**	1.2 (0.6, 2.6); *p* = 0.59	1.0 (0.4, 2.3); *p* = 0.98
	Adhering to two guidelines			
Adhering to sedentary behavior and sleep duration guidelines (REF)				
Not adhering to sedentary behavior and sleep duration guidelines	2.2 (1.1, 4.3); p = **0.02**	2.1 (1.1, 4.2); p **= 0.03**	1.6 (0.9, 2.9); *p* = 0.13	1.5 (0.8, 2.9); *p* = 0.17
Adhering to sedentary behavior and MVPA guidelines (REF)				
Not adhering to sedentary behavior and MVPA guidelines	1.6 (0.8, 3.0); *p* = 0.18	1.3 (0.7, 2.7); p = **0.43**	0.6 (0.3, 1.0); *p* = 0.08	0.5 (0.3, 1.0); *p* = 0.05
Adhering to sleep duration and MVPA guidelines (REF)				
Not adhering to sleep duration and MVPA guidelines	3.3 (1.5, 7.1); p = **0.003**	2.7 (1.2, 6.3); p = **0.02**	1.4 (0.7, 2.9); *p* = 0.41	1.1 (0.5, 2.6); *p* = 0.73
	Adhering to individual guidelines			
Adhering to sleep duration guideline (REF)				
Not adhering to sleep duration guideline	2.3 (1.2, 4.4); p = **0.01**	2.1 (1.1, 4.2); p = **0.03**	2.2 (1.2, 3.9); p = **0.007**	2.0 (1.1, 3.7); p = **0.03**
Adhering to sedentary behavior guideline (REF)				
Not adhering to sedentary behavior guideline	2.3 (0.9, 5.4); *p* = 0.06	2.1 (0.8, 5.4); *p* = 0.10	0.6 (0.3, 1.3); *p* = 0.20	0.7 (0.3, 1.6); *p* = 0.40
Adhering to MVPA guideline (REF)				
Not adhering to MVPA guideline	1.7 (0.9, 3.3); *p* = 0.10	1.4 (0.7, 2.7); *p* = 0.30	0.6 (0.3, 1.2); *p* = 0.16	0.6 (0.3, 1.1); *p* = 0.08

Model 1: unadjusted; Model 2: adjusted for age, race, marital status, education level, poverty income ratio, smoking history, BMI as well as target movement behaviors that are not the exposure of focus (i.e. for sleep + MVPA guidelines, we further adjusted for sedentary behavior); ABBR: MVPA: moderate-vigorous physical activity; REF: reference; **Note: Boldface indicates statistical significance (***p* < *0.05)*

## Discussion

The present study among a nationally representative sample of older adults from the United States found that non-adherence to the 24-HMB guidelines was associated with two-fold higher odds of depression. When examining associations between two movement behavior guidelines, non-adherence to guidelines that included sleep duration combined with either sedentary behavior or MVPA were linked to higher odds of depression. A similar pattern was observed when we examined non-adherence to the individual sleep duration guidelines. Stratified results revealed that many of these associations only remained statistically significant in males, albeit the direction of the association was similar for females, just not statistically significant. However, a potentially detrimental link was observed in females, as adherence to sedentary behavior and MVPA guidelines appeared to be marginally associated with higher odds of depression.

 Among the entire sample, we found that non-adherence to all three 24-HMB guidelines was associated with a two-folder higher odds of depression, this finding generally aligns with existing studies among predominantly Asian populations [21, 36] that have utilized the 24-HMB framework to explore how MVPA, sedentary behavior, and sleep duration guidelines are associated with mental health outcomes, such as depression. For example, a recent study among older adults in China’s Hubei province demonstrated that adhering to all three guidelines was significantly associated with lower PHQ-9 scores, indicating reduced depression severity. Similar analyses in adolescent populations have shown effects consistent with this finding [36–38].

The present study also revealed significant associations between non-adherence to sleep duration and sedentary behavior, as well as sleep duration and MVPA guidelines with higher odds of depression. Existing research, although not always utilizing the 24-HMB framework, supports our current findings. For example, studies among European adults [39] and midlife adults from the US [40] have linked physical inactivity and poor sleep quality to increased depressive symptoms. Moreover, although previous research has emphasized the synergies between adhering to multiple 24-HMB guidelines, our results suggest that meeting physical activity and sedentary behavior guidelines, either alone or in combination, may not produce significant outcomes unless sleep duration is adequately addressed. This is particularly evident in females, where the effects of physical activity and sedentary behavior adherence on mental health were not as prominent when sleep duration was not prioritized.

When we examined individual movement behaviors in relation to depression risk, results showed that adherence to the sleep duration guideline for older adults was significantly associated with lower odds of depression. This finding aligns with existing evidence suggesting that meeting sleep duration recommendations set by the American Academy of Sleep Medicine [41] is linked to reduced depressive symptoms [12, 13, 42]. However, this connection may not be consistently reflected in the current 24-HMB literature. For example, in a secondary analysis conducted among a population from the Netherlands, reallocating time from sleep duration or sedentary behavior to daily MVPA was associated with decreased depressive symptoms, indicating the potential benefits of physical activity, in particular for mental health [43]. While prior studies that have used the 24-HMB framework have found no significant effects of sleep duration but observed significant impacts of MVPA or sedentary behavior, the methodology of treating sleep duration as a continuous variable, rather than binary as the present study did, may reduce the possibility of capturing non-linear relationships with depressive symptoms. Indeed, previous studies have linked sleep duration to depression [44–46] and underscore the potential significance of such non-linear relationships with respect to the 24-HMB for older adults’ mental health. Overall, present study findings provide evidence supporting that adhering to sleep duration guidelines may be more strongly associated to depression risk in older adults, versus the association between adhering to MVPA and sedentary behavior guidelines and depression risk. In light of this being a cross-sectional study that lacks temporality, we posit that sufficient sleep duration may be more effective in reducing depression symptoms, especially when other lifestyle factors, such as physical activity or sedentary behavior, are not prioritized to the same extent.

It is critical to address the sex differences observed in the present study, which indicate significantly higher odds of depression associated with non-adherence to the 24-HMB in older males. However, we also found a potentially detrimental link among older females, as adherence to specific combinations of guidelines, such as sedentary behavior and MVPA was marginally associated with higher odds of depression. While these differences remain unclear, several factors may help explain this potential detrimental association in older females. For example, societal expectations, such as caregiving roles, or occupational responsibilities may shape how older females engage with physical activity and sedentary behavior [8, 47, 48]. These actions could potentially lead to patterns that meet guideline thresholds, while lacking the vital psychological benefits of leisure-based activities [49, 50]. Further, biological and psychological stressors specific to older females may exacerbate depressive symptoms [51], potentially creating adverse associations even when movement behavior guidelines are met. Moreover, the validity of the present study findings may reflect measurement limitations, as adherence to the guidelines may not fully capture qualitative or contextual differences in movement behaviors, such as the nature or purpose of the activities [8, 47, 48]. Additionally, depressive symptoms themselves could influence self-reported physical activity or sedentary behavior, introducing potential reporting bias. As previously mentioned, older females may be more likely to meet activity guidelines through non-leisure activities, like caregiving or occupational tasks, which may not confer the same mental health benefits as leisure-time activities [49, 50]. Similarly, sedentary behavior guidelines may fail to account for the quality of sedentary time, such as whether it involves relaxation or caregiving responsibilities [52], which could influence its association with depression. Residual confounding by factors such as the stress of being a careger, cultural expectations, or social roles may also disproportionately impact older females, contributing to the observed detrimental association between adhering to MVPA and sedentary behavior guidelines and a higher odds of depression. Finally, it is plausible that societal, psychological, and/or physiological differences may modify how adherence to movement behavior guidelines impacts depression in older females. Future research should explore these pathways to better understand the mechanisms underlying this potential detrimental association and the broader contextual factors influencing mental health outcomes across sexes.

In discussing reasons for the observed sex differences, it is essential to distinguish between potential confounding and underlying mechanisms of action. While chronic conditions may influence physical activity levels and depression risk, they are more likely to act as confounders [53] rather than direct mechanisms underlying the observed sex-specific association. A plausible mechanism that could help explain the observed sex differences relates to reductions in physical activity disproportionately impacting male mental health through sex-specific differences in physiological stress responses, such as altered hypothalamic-pituitary-adrenal (HPA) axis activity or increased inflammatory processes, which have been shown to contribute to mental health risk [54, 55]. Additionally, males may have fewer protective psychosocial resources, such as social support, to buffer the impact of reduced activity levels, further exacerbating depression risk. The presence of psychosocial stressors may also differentially impact males and females [56, 57], especially if physical activity is used as a coping mechanism. These factors highlight the complex relationship between sex, movement behaviors, and mental health risk within the older adult demographic. Finally, it is imperative to highlight that while we did not observe as many statistically significant associations between non-adherence to movement behavior guidelines and depression risk among females, our estimates and confidence intervals suggest that the observed associations could be mixed. We caution against over-interpretation of the present study findings based solely on statistical significance and encourage a more nuanced understanding of how 24-h movement behaviors may be related to mental health outcomes within this demographic.

The statistically significant associations observed for the groups that met two or more of the guidelines may have implications for the primary prevention or treatment of depression in older adults. Existing studies on physical activity interventions in older adults have often, though not always, found associations with reduced depressive symptoms in the short- or medium-term [58, 59]. Present study findings suggest the importance of integrating information about sedentary and sleep duration behaviors alongside behavioral or clinical interventions promoting physical activity to prevent or reduce depressive symptoms in older adults. In particular, given the finding that the combination of MVPA and sleep duration decreased the odds of depression, future interventions aimed at promoting mental health in older adults should consider integrating physical activity with non-pharmacological sleep interventions to achieve more clinically significant and lasting results [60, 61]. In addition, the sex differences observed in this study suggest that older males may benefit from interventions targeting multiple movement behaviors at the same time. Moreover, results from the present study raise the question of whether adherence to all three movement behavior guidelines is necessary for optimal mental health in older adulthood, or if prioritizing sleep duration alone could be sufficient Future research should explore whether focusing on a single behavior, such as sleep duration, might be more feasible and effective for certain populations.

Discussing the nuance between adherence and non-adherence to movement behavior guidelines is vital. A minimal deviation via spending fewer or more minutes of MVPA or sedentary behavior could determine whether a participant was classified as either adherent or non-adherent to specific guidelines. Thus, using a cut-off may have implications for how we interpret health outcomes and disease risk, in this case, depression. Moreover, using a binary approach to adherence may mask what may be occurring to participants who are marginally below thresholds (i.e., classified as non-adhering) yet may still be experiencing the same health benefits as those who are adhering. Thus, when examining associations with depression, particularly in older adults, it is important to consider how adherence is being operationalized and the potential limitations related to using this approach. However, one benefit of using the 24-HMB framework was that we examined all three movement behaviors, acknowledging the reality that many older adults are likely to meet only some of the guidelines rather than all three at once. By analyzing these combinations, we were able to capture a more comprehensive picture of the participants’ overall movement behavior patterns and their relation to depression risk.

### Strengths and limitations

The strengths of the present study include the use of a large, nationally representative and diverse sample of older adults from the US. Another strength of this study was the ability to examine all three movement behaviors in relation to depression among a demographic that may be at greater risk of experiencing depression. Further, the large sample size allowed us to examine sex-differences in said associations. However, our study is not without limitations. First, the cross-sectional study design limits our ability to establish temporality and infer causal relationships, meaning we were not able to determien whether movement behaviors predicted depression or vice versa. Second, we used self-reported data to estimate all three movement behaviors along with presence of depression; however, all questionnaires used in the present study have been validated and are known to be reliable for the older adult population. Third, it is possible that there could have been unmeasured confounding that we were unable to account for, such as the use of sleep medications, medical history, or presence of chronic conditions that could be related to both the exposures and outcome, which may have influenced the observed associations. Finally, as previously mentioned in the discussion, the distinction between adherence and non-adherence to movement behavior guidelines is nuanced, as minor deviations can significantly influence health outcomes like depression. However, our analysis included combinations of physical activity, sedentary behavior, and sleep duration, offering a more comprehensive view of lifestyle patterns and their potential protective effects on depression.

## Conclusions

The present study sheds light on the complex relationships between sleep duration, sedentary behavior, and moderate-vigorous physical activity, emphasizing the value of using a framework like the 24-hour movement behavior guidelines to explore how these behaviors relate to the risk of depression among older adults. Our main findings suggest that focusing only on MVPA and sedentary behavior, without considering sleep duration, may be less effective in addressing depression among older adults. The higher odds of depression observed in older males who did not adhere to movement behavior guidelines highlight the importance of considering sex when developing interventions. Future research should explore the interactions between 24-hour movement behaviors and assess whether focusing on one behavior, such as sleep duration, is more feasible and effective for populations at risk of depression.

## Data Availability

No datasets were generated or analysed during the current study.
